# Health care issues in Croatian elections 2005-2009: series of public opinion surveys

**DOI:** 10.3325/cmj.2011.52.585

**Published:** 2011-10

**Authors:** Dagmar Radin, Aleksandar Džakula, Vanesa Benković

**Affiliations:** 1 Department of Political Science and Public Administration Mississippi State University, Mississippi State, Miss, USA; 2 Andrija Štampar School of Public Health School of Medicine University of Zagreb, Zagreb, Croatia; 3 Croatian Society for Pharmacoeconomics and Health Economics, Zagreb, Croatia

## Abstract

**Aim:**

To compare the results of a series of public opinion surveys on experiences with the health care sector in Croatia conducted in the time of elections and to analyze whether political party affiliation had any influence on issues of priority ranking.

**Methods:**

The surveys were conducted during 2005, 2007, and 2009. They were administered through a Computer Assisted Telephone Interviewing method to representative samples of Croatian population and were statistically weighted according to sex, age, level of education, and political party affiliation. The random sampling of the person within the household was done using the table of random numbers.

**Results:**

Health and health care system was the most important issue (58%) during the 2007 parliamentary election and the second most important issue during the 2005 and 2009 elections (46% and 28%). In the 2007 election, health care was viewed as most important by women, respondents with lower education levels, and respondents with lower income. In 2005, the most important health care issues were corruption and lack of funding (45% and 43%, respectively), in 2007 poor organization and lack of funding (43% and 42%, respectively), and in 2009 lack of funding and corruption (51% and 45%, respectively).

**Conclusion:**

Health and health care system were consistently among the top two issues in all elections from 2005 to 2009. The top three most important health care sector issues were corruption, poor organization, and lack of funding. This indicates that political parties should include solutions to these issues in their health care policymaking.

There are various predictors of citizens’ satisfaction with health care system, but there are two main political factors: patient participation and institutional influence of their representative organizations, and political party affiliations (1-4).

Some authors concluded that socio-demographic characteristics were only a minor predictor of satisfaction with health care system, but older age appeared to be one of the most consistent positive determinants of health care satisfaction (5-13). Women were found to be less satisfied with care than men because they were more frequent users of care and had higher expectations (14,15).

Studies in post-communist states provide mixed evidence in this regard. In a 1991 cross-country survey study, most of the respondents believed that, while the market system was essential to economic development, policies that promoted social and economic egalitarianism were important (16). They also found that women, those with lower educational level, and those with lower income were more likely to be supportive of socialist principles. On the other hand, a study conducted in 1999 showed a change in value systems, with a majority of respondents from Poland and Hungary not favoring further redistribution of income, even at the expense of welfare (17). Furthermore, there was a positive correlation between education and positive attitude to health care competition. Still, in Hungary the older generations were predominantly against increases in health care competition.

Since the early 1990s, the public level of satisfaction with the health care system reforms in Croatia has not matched the apparent success of the reform goals. A 1994 consumer survey found that a vast majority of lower- and middle-income Croats was skeptical of health care reforms (18). They attributed this to the legacy of universal access to health care, the view of health care access as a universal right, negative consequences on the patients, and lack of public understanding of the reforms.

The same study (18) found the distribution of out-of-pocket payments and co-payment for health care to represent a regressive burden on those in the lower income group. Patient satisfaction was also low: 44% were dissatisfied with the quality of health facilities and 48% with the equipment (18). Similar face-to-face interviews found that citizens of Croatia did not hold a positive view of the health insurance reform (19). Their primary concern were limitations of their rights and the increase in the financial burden related to medical care. A 2005 survey found that during the presidential election campaign period health care was the second most important issue on the voters’ minds, closely following economy (20).

Finally, a national survey on patients’ satisfaction with hospital and primary health care in 2006 showed considerable concerns with relations between patients and medical professionals, hospital accommodation, communication between primary and secondary health care, and corruption (21,22).

In this study, we present and compare the results of a series of public opinions surveys conducted between 2005 and 2009. This is the first study that targeted public experiences and voters’ opinion as an evaluation of the health care sector in Croatia.

## Methods

The surveys were administered during 2005, 2007, and 2009 through a Computer Assisted Telephone Interviewing method (CATI) to a representative sample of the Croatian population and were statistically weighted according to sex, age, level of education, and political party affiliation. The elections involved were the presidential election in 2005, parliamentary election in 2007, and the presidential election in 2009. The research in 2005 involved 1000 respondents, in 2007 it involved 1500, and in 2009 it involved 800 respondents. The sampling error was estimated using population size and the standard deviation of our sample and the maximum sample error was ±2.5% to 3.2%. All of the surveys were administered as election surveys with health-related questions appended (web-extra material)(web extra material 1).

The probabilistic sample was stratified in two stages with the following characteristics: six traditional regions – City of Zagreb region, Northern, Southern, Central, Western, and Eastern region, defined through existing counties (to achieve sorting election units based on principle of exclusiveness and exhaustiveness), and according to settlement size. Unit allocations over strata were performed proportionally to strata size (number of adult examinees in stratum). The urbanization level was distributed in 4 population size categories (up to 2000 residents, 2001-10 000, 10 001-100 000, and more than 100 000 residents). Randomization of the sample was computer-based according to stratum definition, but the sample was additionally weighted to obtain a fully representative sample of the Croatian voting body.

Random sampling of persons within households was done using the table of random numbers. Studies have shown that this type of statistical adjusting of data obtained through CATI was appropriate taking into account that the survey was conducted during a short period (21-24). The questionnaires were divided into three question groups. The first question group consisted of general questions on household size, location, and region. The second group of questions was related to the respondent’s age, sex, employment status, and income. The third question group consisted of questions related to the elections, presidential and parliamentary role in the interior and foreign policy, health care issues, and health priorities related to the president’s/parliament`s work. Questions were made on the basis of topics recognized in the press as the most important in the period of two months before the election (20).

While the raw data for presidential election included individual candidates and their party affiliation, we were interested in how public opinion may be affected by differences between the two major political options. Thus, we created a new variable that grouped the central-right (C-R) parties/party coalitions vs central-left (C-L) parties/party coalitions. Candidates’ ideological standings were assessed by a qualitative analysis of party platforms (if candidates were affiliated) or public campaign statements (independent candidates). The information was gathered from the respective party/candidate internet homepages (25,26) as well as from the Comparative Manifesto Project Database (27).

Out of the twelve candidates in the survey, four were C-L (Milan Bandić, Ivo Josipović, Damir Kajin, and Vesna Pusić) and eight were C-R (Andrija Hebrang, Josip Jurčević, Boris Mikšić, Dragan Primorac, Vesna Škare Ozbolt, Miroslav Tuđman, Nadan Vidošević, and Slavko Vukšić).

### Statistical analysis

Bivariate analysis was performed with χ^2^ test. Multivariable model, controlling for respondent’s age, sex, and place of residence was used to determine the difference between left- and right-wing voters’ opinions and was expressed as odds ratio with 95% confidence interval. The analysis was performed by SPSS, version 12.0 and 16.0 (SPSS Inc., Chicago, IL, USA), with statistical significance level set at *P* < 0.05.

## Results

The results presented in this section are not exhaustive of all the questions asked in the individual public opinion surveys. The purpose of this study was to compare the answers to the same question asked across all surveys and analyze specific determinants relevant for public opinion during election. Thus, we divided the results into sections*:* general election comparison, socioeconomic factors, and party affiliations.

### General election comparison

Health and health care system were placed near the top of the list of important issues in Croatian elections since 2005 (Table 1). In the 2005 and 2009 presidential election, health care was the second most important issue (46% and 28%, respectively) and in the 2007 parliamentary election the most important issue (58%), closely followed by economy (57%). When asked about the most important problems in the health care sector, the most highly ranked were corruption, lack of funding, and poor organization.

**Table 1:** Comparison of 2005-2009 Croatian pre-election survey responses. Highest values in each group are in bold

	Percentage of voters in		
Question	2005 presidential election*	2007 parliamentary election	2009 presidential election
One of the two most important issues in Croatia:			
Foreign policy	12	8	23
health and health care system	**46**	**58**	**28**
economy	**70**	**57**	**78**
domestic policy	13	5	8
social care	18	25	**28**
education	26	30	20
One of the two most important health care problems:			
corruption	45	40	45
poor organization	41	43	44
lack of funding	43	42	51
suboptimal performance	13	14	8
insufficient equipment	30	32	39
other/not responded/do not know	10	10	14
Number of respondents	1000	1500	800

*This information was in part taken from Džakula et al (20).

In 2005, the two most important health care issues were corruption and lack of funding (45% and 43%, respectively), in 2007 poor organization and lack of funding (43% and 42%, respectively) and in 2009 lack of funding and corruption (51% and 45%, respectively).

Regarding the order of priority in which the newly elected government should address the existing issues, health care ranked very low (fifth both in 2007 and 2009), with corruption, unemployment, and economy ranking higher.

### Socio-economic determinants

We also determined which socio-economic factors affected individuals’ view of health care as the most important issue. For this analysis, we selected the 2007 election because then health care was recognized as the top priority by the majority of the respondents. A total of 65.3% of female respondents viewed health care as the most important issue. Respondents with primary and secondary education and those who were part-time employed or unemployed also viewed health care as the most important issue. Respondents in the low and low-middle income group and C-R voters preferred private care and viewed poor organization, lack of funding, poor performance, and insufficient equipment as most problematic in the health care system. They were also more likely to choose health care as the most important issue (Table 2).

**Table 2:** Proportions of responses to the most important issue in 2007 election

	Percentage of respondents who considered the most important issue to be	
Characteristic	health care	other issues
Sex:		
male	48.9	51
female*	65.3	34.7
Education level:		
no primary	51.2	48.8
primary*	67	33
secondary*	55	44.4
university	45	54.6
Employment status:		
employed	51.6	48.3
farming	43.7	56.2
part time/unemployed*	61.5	38.4
Household Income (HRK):		
up to 1000	57.4	42.6
1001-2500*	64.5	35.5
2501-4000*	65.2	34.8
4001-5500*	59.6	40
5501-7000	5.7	48.24
7001-8500	49.7	50
8501+	44	56
do not know/not available	58.6	41.4
Settlement size:		
1-2000	51.8	48.2
2001-10 000*	62.6	37.4
10 001-100 000	53.3	46.7
100 001+*	60.4	39.6
Top health care problem to be addressed:		
poor organization*	60.9	39
lack of funding*	57.5	42.4
poor performance of medical staff*	64.7	35.2
inadequate equipment*	59	41
corruption	53.4	46.4
other	62.3	37.7
Party identification:		
center-left	55	45
center-right*	60.3	39
Preference of treatment:		
private*	62	38
public*	56	44
Reason for private care visit:		
never visited*	58.4	41.6
long lines in public health facilities*	59.3	40.7
better quality of care than public	58	42
public physician referral to private sector	44.7	55.3
private physician is family member	53.2	46.8
do not know/not available	48.3	51.7

*t test was performed to assess the differences in probabilities; significance level is at *P* < 0.05.

### Party affiliations

In 2005, significantly more C-R respondents than their C-L counterparts viewed health care as most important (26.9% vs 14.8%, *P* < 0.05), while in 2007 and 2009 the difference was no longer significant. Significantly more C-L respondents viewed economy as most important (53.9% vs 40.0%, *P* < 0.05), but in 2007 and 2009 this difference was also no longer significant. When asked about specific health care sector problems, in 2005 more C-L respondents considered suboptimal physician performance as most important (6.4% vs 4.5%) and more C-R respondents considered lack of funding (31.0% vs 20.5%). In 2007, more C-R respondents considered insufficient equipment as most important (33.1% vs 43.6%), while more C-L respondents considered poor organization (51.2% vs 45.9%). In 2009, both C-L and C-R respondents considered lack of funding as most important (47.8% and 56.4%) (Table 3).

**Table 3:** Comparison of 2005 and 2007 Croatian pre-election survey responses. Highest values in each group are in bold

	Percentage of respondents					
Question	2005 presidential election	2007 parliamentary election	party affiliation 2005*		party affiliation 2007	
			center left	center-right	center left	center-right
Among two most important issues in Croatia:						
health and health care system	13	5	6.4	8.3	9.9	11.9
foreign policy	12	8	5.9	5.8	5.9^†^	10.3^†^
economy	**70**	**57**	**53.9^†^**	**40.0^†^**	**65.8**	**60.1**
domestic politics	**46**	**58**	**14.8^‡^**	**26.9^‡^**	**62.2**	**60.3**
social care	18	25	7.9	5.8	25.6	21.4
education	26	30	8.8	6.4	30.5	38.2
Among two most important health care problems:						
corruption	**45**	40	**25.6**	**20.6**	**49.8**	43.8
poor organization	41	**43**	**26.2**	20.0	**51.2**	**45.9**
lack of funding	**43**	**42**	20.5	**31.0**	44.4	**47.3**
suboptimal physician performance	13	14	6.4^†^	4.5^†^	14.2	14.9
insufficient equipment	30	32	15.9	15.5	33.1^†^	43.6^†^
other/not responded/do not know	10	10	9.0	10.9	7.2	4.2
Priority of government post election:						
corruption	**61**	**43**	**78.3**	**70.8**	**52.6^‡^**	**40.1^‡^**
national budget	**44**	1	**53**	51.5	1.1	2.8
EU accession	24	7	32.6	25.1	7.7	9.0
relations with the International Criminal Tribunal for former Yugoslavia	14	12	15.6	**96.1**	7.1^‡^	25.8^‡^
economy	4	32	2.6	4.5	40.3	29.2
unemployment	4	**43**	3.2	3	**41.8^‡^**	**48.8^‡^**
foreign policy	1	4	0.9^†^	1.7†	4.7	2.5
domestic policy	1	21	1.1	0.7	24.1^‡^	18.5^‡^
secret intelligence^§^	7		7.6	9.4		
health care^║^		19			17.1	21.3
Number of respondents	1000	1500				

*This information was in part taken from Dzakula et al (20).

†F test was performed to assess the differences in probabilities. Significance level is at *P* > 0.05.

‡Significance level is at *P* > 0.001.

§This response option was not included in the 2007 questionnaire.

║This response option was not included in the 2005 questionnaire.

### Ranking health care priorities – 2009 presidential election and party affiliation

As two most important health care problems in Croatia, respondents identified corruption (about 44.8%) and lack of funding (50.5%), similar to the findings in 2005 and 2007. With regard to poor organization and insufficient equipment, more C-L respondents than their C-R counterparts considered both issues most important (45.1% vs 41.3% for poor organization and 38.9% vs 38.6% for insufficient equipment). More C-R than C-L respondents considered lack of funding and suboptimal physician`s performance most important (56.4% vs 47.8% and 7.8% vs 7.4%, respectively) (Table 4).

**Table 4:** Health care related opinion in the 2009 Croatian presidential election campaign. Highest values in each group are in bold

	Percentage of respondents		
Question	total	party affiliation	
One of the two most important health care problems:		center left	center right
corruption	**44.8**	**46.1**	**42.9**
poor organization*	44.3	45.1	41.3
lack of funding	**50.5**	**47.8**	**56.4**
insufficient equipment*	39.4	38.9	38.6
suboptimal physicians’ performance	7.6	7.4	7.8
other/not responded/do not know	14.0	14.7	13.1
Experience with health care problem in the last year:			
corruption*	**8.4**	8.1	**8.9**
poor organization*	**20.4**	**22.3**	**17.4**
lack of funding^†^	6.9	**76.6**	7.3
suboptimal physicians’ performance	5.7	4.9	6.9
insufficient equipment	5.7	5.9	5.4
other/not responded/do not know	52.9	52.2	52.9
Post election priority in health care:			
corruption*	**33.0**	**32.4**	**34.0**
poor organization*	**22.3**	**25.7**	17.0
lack of funding^†^	17.8	17.4	**18.5**
insufficient equipment	8.1	6.9	10.0
suboptimal physicians’ performance	2.7	3.2	1.9
other/not responded/do not know	16.1	14.4	18.6
Number of respondents	667	408	259

*t test was performed to assess the differences in probabilities; significance level is at *P* < 0.001.

†t test was performed to assess the differences in probabilities; significance level is at *P* < 0.05.

When asked about experiences with a health care problem in the previous year, C-L respondents selected poor organization (22.3%), lack of funding (7.3%), and health care corruption (8.9%). C-R respondents also reported experience with these three issues, but in different order – 17.4% experienced poor organization, 8.4% corruption, and 6.9% lack of funding. As a priority after election, C-L voters predominantly chose corruption and poor organization, while C-R voters chose corruption and lack of funding. Looking at the number of visits to the private practice, nearly 60% of respondents who visited a private practice did so only once, while almost 11% visited it five or more times. More C-L respondents visited private practice once (39.1% vs 20.3). The primary reasons for visiting a private practice were long waiting lines in the public sector and better quality of care provided.

## Discussion

Our study showed that voters in Croatian elections viewed health and health care as important issues. Health care consistently ranked among the top issues in Croatian elections, with the exception of the last election when the economic and financial consequences of the world crises overshadowed it. The top three health care issues in all of the elections were corruption, poor organization, and lack of funding. The persistence of the same issues in the health care sector over the years indicates that they remain ineffectively addressed by the government. The results presented here were obtained in a telephone survey, and therefore are prone to certain levels of selection bias. Furthermore, the comparison across elections was not as extensive as wanted given the limited number of comparable questions and their diversity.

Despite the reforms that have been implemented in Croatia over the years, and most notably the sweeping changes aimed at cost-cutting in the last year, not much has been done to alleviate public concerns. As an example, while one of the aims of the 2009 reforms was to shorten waiting lines, the most often cited reason for using private health providers in that same year was the long waiting lines in the public health care system (28-30).

We kept observing a counterintuitive finding that while the public perceived health care as one of the most problem-ridden sectors, it did not view it as a post-election priority. One possible explanation is that the issue of reforming a health care system is a complex one, which requires detailed information and specialized knowledge (31). In fact, there is no clear blueprint for reforming a health care system successfully compared to a blueprint followed for enacting macroeconomic reforms in the 1990s. Another explanation is that there have been other issues of equal importance (unemployment, the sluggish economy) in Croatia since the transition.

In the 2009 presidential election, health care lost priority as Croatia was facing the consequences of the international financial and economic crises. Considering long term structural problems in the Croatian economy unresolved since the beginning of the economic transition of the early 1990s, it is not surprising that we found that the state of the economy was of pressing concern to most citizens.

Overall there were no significant differences between C-L and C-R voters when it came to identifying health care or particular health care problems as an important issue. However, in 2009 some differences were pronounced, such as the identification of health care problems, experience with problems, and priority that should be given to particular health care problems. Some findings are, therefore, in accordance with the trends in other consolidated democracies where left oriented voters are more supportive of, and concerned with, social welfare policies, including health care (1,2). This also signals to Croatian parties that they can focus on health care issues in order to achieve electoral gain. Our study showed that women, respondents with lower education levels, and respondents with lower income viewed health care as the most important issue in the 2007 election. A 2007 study found that only sex was a significant predictor of attitudes toward selecting health care as the most important issue (32). This confirms that the most socially and economically vulnerable groups in Croatian society are the ones that have the greatest concern for health care, which brings up the issue of health inequalities. In the 2009 presidential election, many of these differences disappeared as the government, public, and media focus shifted toward problems in the domestic economy brought on or accentuated by the global economic and financial crises.

Finally, health care corruption is a persistent problem in Croatian health care, which is not only a product of public perception, but is supported by public experiences with corruption.

We demonstrated that health care was an issue that cut across party lines. However, until recently, only two Croatian parties had a clearly stated mandate that included health care sector performance and reforms. This discrepancy between public opinion and party mandates indicates a greater need for the political parties to compete on the basis of clearly articulated issue-based programs, and include public concerns in their health care policymaking.
