# Supplementary material for: Health care issues in Croatian elections 2005-2009: series of public opinion surveys
Source: Croat Med J. 2011 Oct;52(5):585–92. doi: 10.3325/cmj.2011.52.585 (PMC3195967; doi:10.3325/cmj.2011.52.585)
Supplement: Supplementary material 1 [file CroatMedJ_52_s001.pdf]

IZBORI I ZDRAVLJE - 2005

Agencija Puls provela je telefonsko istraživanje 28. i 29. prosinca 2004. na nacionalno-reprezentativnom uzorku od 1000 ispitanika starijih od 18 godina. U sklopu istraživanja postavili smo nekoliko pitanja koja su vezana uz zdravstvo.

1. **Koja dva od ovih područja su po Vašem mišljenju najvažnija za Hrvatsku i njezine građane u ovom trenutku?**
2. **Koja dva od ovih područja su po Vašem mišljenju najvažnija za Hrvatsku i njezine građane u ovom trenutku? – prema preferencijama predsjedničkih kandidata**
3. **S koja dva problema bi se, prema Vašem mišljenju, Predsjednik Hrvatske trebao najviše baviti?**
4. **S koja dva problema bi se, prema Vašem mišljenju, Predsjednik Hrvatske trebao najviše baviti? – prema preferencijama predsjedničkih kandidata**
5. **Koja su dva najveća problema u zdravstvenoj zaštiti građana u Hrvatskoj?**
6. **Koja su dva najveća problema u zdravstvenoj zaštiti građana u Hrvatskoj? – prema preferencijama predsjedničkih kandidata**
7. **S koja dva problema iz područja zdravstva bi se trebao najviše baviti budući Predsjednik Hrvatske?**
8. **S koja dva problema iz područja zdravstva bi se trebao najviše baviti budući Predsjednik Hrvatske? – prema preferencijama predsjedničkih kandidata**

UPITNIK 2007

P1. Koje dva od ovih područja su po Vašem mišljenju najvažnija za nadolazeće Parlamentarne izbore? PROČITAJ

1. Vanjska Politika
2. Gospodarstvo
3. Zdravlje I zdravstvena zaštita
4. Politički odnosi u Republici Hrvatskoj
5. Socijalna zaštita
6. Školstvo

7. Drugo: navedi ____________________
8. Ne zna (NE ČITAJ)

P2. S koja dva problema bi se, prema vašem mišljenju, parlament Republike Hrvatske trebao prvo suočiti nakon 2007 izbora? PROČITAJ

1. Odnosi s Haškim sudom
2. Pridruživanje EU
3. Unutarnja politika/unutarnji problemi
4. Korupcija
5. Gospodarstvo
6. Zdravstvo
7. Rad tajnih službi
8. Državni proračun
9. Nezaposlenost
10. Nešto drugo
11. Vanjska politika
12. Nešto drugo. Što? _______ (NE ČITAJ)

98. Ne zna (NE ČITAJ)

P3. S kojim problemom bi se, prema vaše mišljenu, parlament Republike Hrvatske trebao suočiti kao posljednje, nakon izbora 2007? PROČITAJ; JEDNA ODGOVOR

1. Odnosi s Haškim sudom
2. Pridruživanje EU
3. Unutarnja politika/unutarnji problemi
4. Korupcija
5. Gospodarstvo
6. Zdravstvo
7. Rad tajnih službi
8. Državni proračun
9. Nezaposlenost
10. Vanjska politika
11. Nešto drugo. Što? _______ (NE ČITAJ)

98. Ne zna (NE ČITAJ)

P4. Koja su dva najveća problema danas u zdravstvenoj zaštiti građana? PROČITAJ

1. Loša organizacija zdravstvenog sustava
2. Nedostatak novca
3. Neadekvatan rad zdravstvenih radnika
4. Loša oprema i prostori
5. Korupcija
6. Nešto drugo:___________________
7. Ništa od navedenog (NE ČITAJ)
8. NZ/BO (NE ČITAJ)

P5. S kojim ste se problemom u zdravstvu najčešće osobno suočili prošle godine (navedi sve sto je)? PROČITAJ; MOGUĆE VIŠE ODGOVORA

1. Loša organizacija zdravstvenog sustava
2. Nedostatak novca

3. Neadekvatan rad zdravstvenih radnika
4. Loša oprema i prostori
5. Korupcija
6. Nešto drugo:______________________
7. Ništa od navedenog
8. NZ/BO

P6. S kojim bi se problemom u zdravstvu Parlament Republike Hrvatske trebao suočiti kao prvo, nakon 2007 izbora?
1. Loša organizacija zdravstvenog sustava
2. Nedostatak novca
3. Neadekvatan rad zdravstvenih radnika
4. Loša oprema i prostori
5. Korupcija
6. Nešto drugo:______________________
7. Ništa od navedenog
8. NZ/BO

P7. Koliko ste puta u zadnjih godinu dana odabrali posjetu liječniku u privatnoj praksi/privatnoj klinici?

____

P8. Koji je bio primarni razlog vašoj posjeti liječniku privatniku/privatnoj klinici prošle godine?
1. Nisam nikada bio/bila
2. Predugo čekanje u javnom zdravstvu za pretrage, itd.
3. Bolja kvaliteta zdravstvene usluge u odnosu na državne institucije
4. Preporuka liječnika iz javnog zdravstva na posjetu privatniku
5. Privatni liječnik je član obitelji/prijatelj
6. NZ/BO

P9. Pri korištenju zdravstvene zaštite, ako je moguće, preferirate:
1. Privatnu praksu
2. Javne ustanove

P10. Izbori za zastupnike u Hrvatski sabor će se održati u narednu nedjelju, 25. studenog. Hoćete li Vi osobno izaći na te izbore i glasovati? PROČITAJ

1 – Sigurno da
2 – Vjerojatno da
3 – Vjerojatno ne
4 – Sigurno ne
8 – Ne zna

P11. Za koju stranku ili koaliciju ćete najvjerojatnije glasovati ako izađete na te izbore? PROČITAJ; ROTIRAJ

1 - HDSSB (SAMO 4 i 5 IJ) – Branimir Glavaš
2 - HDZ

3 - HNS
4 - HSP
5 - HSS-HSLS-PGS
6 - HSU
7 - Nezavisna lista Tonči Tadić (SAMO U 10 IJ)
8 - SDP
9 - SDSS (SAMO U 9 IJ)
10 - Neka druga. Koja? ______________
-------- NE ČITAJ -----------
11 – Ne znam/Neodlučan → PITAJ P12
12 – Neću glasovati → PITAJ P12
13 – Odbija → PITAJ P12

P12. Bez obzira na sve, sigurno nemate jednake stavove o svim strankama i listama kandidata. Molim Vas da mi kažete koje od stranaka ili koalicija s ove liste je Vama osobno ipak nešto bliža od ostalih?.
PROČITAJ; ROTIRAJ
1 - HDSSB (SAMO 4 i 5 IJ) – Branimir Glavaš
2 - HDZ
3 - HNS
4 - HSP
5 - HSS-HSLS-PGS
6 - HSU
7 - Nezavisna lista Tonči Tadić (SAMO U 10 IJ)
8 - SDP
9 - SDSS (SAMO U 9 IJ)
10 - Neka druga. Koja? ______________
-------- NE ČITAJ -----------
11 – Ne znam/Neodlučan
12 – Neću glasovati
13 – Odbija

P13. Osim glasanja, koliko često ste uključeni u druge oblike političkih aktivnosti kao npr. kontakt sa svojim predstavnika u saboru o temama koje me brinu, lijepljenje letaka za izbore, novčana donacija strankama, volonter za vrijeme političkih kampanja, itd.?
PROČITAJ
1. Nikada
2. Rijetko
3. Ponekad
4. Cesto
5. Uvijek
8. Ne znam (NE ČITAJ

ZDRAVSTVENE TEME U PARLAMENTARNIM IZBORIMA 2009
Health care issues in the Parliament elections 2009

0. Sections - Pre- interview administration

a) Identifikacijska oznaka ispitanika

b) Datum intervjua

c) Vrijeme početka

d) Vrijeme završetka

e) Tip Naselja

1. *Grad*
2. *Selo*

f) Veličina naselja

1. *Više od 100 000 stanovnika*
2. *10 000 - 99 999 stanovnika*
3. *2 000 – 9 999 stanovnika*
4. *0 – 1 999 stanovnika*

e) Regija

1) *Zagreb I okolica*
2) *Sjeverna Hrvatska*
3) *Slavonija*
4) *Lika I Banovina*
5) *Istra I Hrvatsko Primorje*
6) *Dalmacija*

SECTIONS:

I. Voting/participation/party identification
II. Health care
III. Socio-demographic characteristics/household economic conditions
IV. Corruption specific issues

I. Glasanje/participacija/

1) Da li ćete izaći na glasanje na predstojećim Predsjedničkim izborima u Hrvatskoj?

1. Sigurno neću izaći
2. Vjerojatno neću izaći

3. Vjerojatno ću izaći
4. Sigurno neću izaći
8. Ne znam

2) Kada bi ovaj vikend bili izbori za koga biste glasovali?

1. MILAN BANDIĆ
2. ANDRIJA HEBRANG
3. IVO JOSIPOVIĆ
4. JOSIP JURČEVIĆ
5. DAMIR KAJIN
6. BORIS MIKŠIĆ
7. DRAGAN PRIMORAC
8. VESNA PUSIĆ
9. VESNA ŠKARE OŽBOLT
10. MIROSLAV TUĐMAN
11. NADAN VIDOŠEVIĆ
12. SLAVKO VUKŠIĆ

3) Osim glasanja, koliko često ste uključeni u druge oblike političkih aktivnosti (npr. Kontaktiram svog predstavnika u saboru o temama koje me brinu, lijepljenje letaka za izbore, novčana donacija strankama, volonter za vrijeme političkih kampanja, itd.)?

1. Nikada
2. Rijetko
3. Ponekad
4. Cesto
5. Uvijek
8. Ne znam

II. ZDRAVSTVO/HEALTH CARE

2) Koje **<u>dva od ovih područja</u>** su po Vašem mišljenju najvažnija za nadolazeće Predsjedničke izbore?

9. Vanjska Politika i pridruživanje EU
10. Gospodarstvo
11. Zdravlje i zdravstvena zaštita
12. Politički odnosi u Republici Hrvatskoj
13. Socijalna zaštita
14. Školstvo

15. Drugo: navedi ____________________
16. Ne zna

3) S **koja dva problema** bi se, prema vašem mišljenju, Predsjednik Republike Hrvatske trebao ***prvo suočiti*** nakon izbora 2010?

13. Odnosi s Haškim sudom
14. Pridruživanje EU
15. Unutarnja politika/unutarnji problemi
16. Korupcija
17. Gospodarstvo
18. Zdravstvo
19. Obrazovanje
20. Socijalna zaštita
21. Nezaposlenost
22. Preustroj Državnog proračuna
23. Nezaposlenost
24. Ne znam

4) S kojim **problemom** bi se, prema vaše mišljenu, Predsjednik Republike Hrvatske trebao ***suočiti kao posljednje***, nakon izbora 2009?

1. Odnosi s Haškim sudom
2. Pridruživanje EU
3. Unutarnja politika/unutarnji problemi
4. Korupcija
5. Gospodarstvo
6. Zdravstvo
7. Obrazovanje
8. Socijalna zaštita
9. Nezaposlenost
10. Preustroj Državnog proračuna
11. Nezaposlenost
12. Ne znam

5) Koja su **dva najveća problema** danas u zdravstvenoj zaštiti građana?

9. Loša organizacija zdravstvenog sustava
10. Nedostatak novca
11. Neadekvatan rad zdravstvenih radnika
12. Loša oprema i prostori
13. Korupcija
14. Nešto drugo:__________________
15. Ništa od navedenog

16. NZ/BO

6) S kojim ste se **problemom** u zdravstvu najčešće osobno suočili prošle godine?

1. Loša organizacija zdravstvenog sustava
2. Nedostatak novca
3. Neadekvatan rad zdravstvenih radnika
4. Loša oprema i prostori
5. Korupcija
6. Nešto drugo:______________________
7. Ništa od navedenog

8. NZ/BO

6) S kojim bi se **problemom** u zdravstvu Predsjednik Republike Hrvatske trebao suočiti kao prvo, nakon 2009. izbora?

9. Loša organizacija zdravstvenog sustava
10. Nedostatak novca
11. Neadekvatan rad zdravstvenih radnika
12. Loša oprema i prostori
13. Korupcija
14. Nešto drugo:______________________
15. Ništa od navedenog
16. NZ/BO

7) Koliko ste puta u zadnjih godinu dana odabrali posjetu liječniku u privatnoj praksi/privatnoj klinici?

---

8) Koji je bio **primarni razlog** vašoj posjeti liječniku privatniku/privatnoj klinici prošle godine?

7. Nisam nikada bio/bila
8. Predugo čekanje u javnom zdravstvu za pretrage, itd.
9. Bolja kvaliteta zdravstvene usluge u odnosu na državne institucije
10. Preporuka liječnika iz javnog zdravstva na posjetu privatniku
11. Privatni liječnik je član obitelji/prijatelj
12. NZ/BO

9) Pri korištenju zdravstvene zaštite, ako je moguće, preferirate:

1. Privatnu praksu
2. Javne ustanove

III. Socio-demographic

1)Dob

2) Spol

a. Muški
b. Ženski

3) Obrazovanje

1. Bez osnovne škole
2. Osnovna škola
3. Srednja škola, gimnazija ili stručna
4. Visa škola i fakultet
5. Magistar/Doktor znanosti
6. NZ/BO

4) Koje je vaše zanimanje?

1. Slobodna profesija
2. Stručnjaci i intelektualci
3. Visi menadžment, visi rukovoditelji, direktori
4. Srednji menadžment
5. Službenici
6. Kvalificirani radnici, uključujući i bolničke sestre
7. Nekvalificirani i niskokvalificirani radnici
8. Poljoprivrednici i ribari
9. Vojska i policija

98. NZ/BO

5) Jeste li vi.....?

1) Zaposlenik
2) Umirovljenik/ca
3) Domaćica
4) Student/cica
5) Radi honorarno, ali ne stalno zaposlen/a
6) Potpuno nezaposlen/a
7) Nešto drugo

NZ/BO

7) Procijenite Vaše imovinsko stanje, odnosno imovinsko stanje Vaše obitelji

1. Puno lošije od većine drugih
2. Nešto lošije od većine drugih
3. Ni bolje ni lošije od većine drugih
4. Nešto bolje od većine drugih
5. Puno bolje od većine drugih

8) Broj stalnih članova kućanstva
